# Fenofibrate Therapy Restores Antioxidant Protection and Improves Myocardial Insulin Resistance in a Rat Model of Metabolic Syndrome and Myocardial Ischemia: The Role of Angiotensin II

**DOI:** 10.3390/molecules22010031

**Published:** 2016-12-28

**Authors:** Luz Ibarra-Lara, María Sánchez-Aguilar, Alicia Sánchez-Mendoza, Leonardo Del Valle-Mondragón, Elizabeth Soria-Castro, Elizabeth Carreón-Torres, Eulises Díaz-Díaz, Héctor Vázquez-Meza, Verónica Guarner-Lans, María Esther Rubio-Ruiz

**Affiliations:** 1 Department of Pharmacology, Juan Badiano 1, Sección XVI, Tlalpan, México City 14080, Mexico; luzibarralara@gmail.com (L.I.-L.); msanchezaguilar@gmail.com (M.S.-A.); masanchez@gmail.com (A.S.-M.); leonardodvm65@hotmail.com (L.D.V.-M.); 2 Department of Pathology, Juan Badiano 1, Sección XVI, Tlalpan, México City 14080, Mexico; sorieli@hotmail.com; 3 Department of Molecular Biology, Juan Badiano 1, Sección XVI, Tlalpan, México City 14080, Mexico; qfbelizabethcm@yahoo.es; 4 Department of Reproductive Biology, Instituto Nacional de Ciencias Médicas y de la Nutrición “Salvador Zubirán”, Vasco de Quiroga 15, Sección XVI, Tlalpan, México City 14000, Mexico; eulisesd@yahoo.com; 5 Departamento de Bioquímica, Facultad de Medicina, Universidad Nacional Autónoma de México (UNAM), Ciudad Universitaria, México City 04510, Mexico; hvazquez@bq.unam.mx; 6 Department of Physiology, Instituto Nacional de Cardiología “Ignacio Chávez”, Juan Badiano 1, Sección XVI, Tlalpan, México City 14080, Mexico; gualanv@yahoo.com

**Keywords:** metabolic syndrome, insulin resistance, myocardial ischemia, fenofibrate, oxidative stress, angiotensin II

## Abstract

Renin-angiotensin system (RAS) activation promotes oxidative stress which increases the risk of cardiac dysfunction in metabolic syndrome (MetS) and favors local insulin resistance. Fibrates regulate RAS improving MetS, type-2 diabetes and cardiovascular diseases. We studied the effect of fenofibrate treatment on the myocardic signaling pathway of Angiotensin II (Ang II)/Angiotensin II type 1 receptor (AT1) and its relationship with oxidative stress and myocardial insulin resistance in MetS rats under heart ischemia. Control and MetS rats were assigned to the following groups: (a) sham; (b) vehicle-treated myocardial infarction (MI) (MI-V); and (c) fenofibrate-treated myocardial infarction (MI-F). Treatment with fenofibrate significantly reduced triglycerides, non-high density lipoprotein cholesterol (non-HDL-C), insulin levels and insulin resistance index (HOMA-IR) in MetS animals. MetS and MI increased Ang II concentration and AT1 expression, favored myocardial oxidative stress (high levels of malondialdehyde, overexpression of nicotinamide adenine dinucleotide phosphate (NADPH) oxidase 4 (NOX4), decreased total antioxidant capacity and diminished expression of superoxide dismutase (SOD)1, SOD2 and catalase) and inhibited expression of the insulin signaling cascade: phosphatidylinositol 3-kinase (PI3K)/protein kinase B (PkB, also known as Akt)/Glut-4/endothelial nitric oxide synthase (eNOS). In conclusion, fenofibrate treatment favors an antioxidant environment as a consequence of a reduction of the Ang II/AT1/NOX4 signaling pathway, reestablishing the cardiac insulin signaling pathway. This might optimize cardiac metabolism and improve the vasodilator function during myocardial ischemia.

## 1. Introduction

Metabolic syndrome (MetS) is a public health problem that results from a complex interaction of factors including genetic predisposition, diet, metabolism and physical activity. Insulin resistance, a characteristic of MetS, impairs the ability of the heart to adjust to changing energy demands. It increases the delivery of fatty acids to the heart and reduces the use of glucose, thereby shifting the metabolism of the heart toward a greater reliance on fatty acids for energy obtainment [1]. Moreover, MetS is accompanied by an increased generation of reactive oxygen species (ROS), lipoperoxidation and increased peroxidation of nitric oxide (NO) to its toxic species, that result in oxidative stress [2].

Myocardial ischemia is followed by functional, biochemical and morphological consequences, including the inhibition of insulin signaling [3,4]. In diabetic patients, impaired myocardial contractility is associated with the loss of insulin effects and mitochondrial dysfunction [5]. Additionally, the deleterious effect of oxidative stress has been demonstrated in cardiac ischemia and reperfusion experiments, both, in vivo and in vitro in a MetS model developed in our institution [6].

Dysregulation of renin-angiotensin system (RAS) is associated with increased cardiovascular risk [7,8]. Angiotensin II (Ang II) is the main effector molecule of RAS; it produces superoxide anion via Angiotensin II type 1 receptor (AT1) and activation of the reduced form of nicotinamide adenine dinucleotide phosphate (NADPH) oxidase (NOX). Additionally, superoxide anion and peroxynitrite interact with NO and oxidize tetrahydrobiopterin (BH_4_). They lead to endothelial nitric oxide synthase (eNOS) uncoupling and therefore to endothelial dysfunction associated with increased oxidative stress, in pathological conditions such as insulin resistance [9,10,11].

Furthermore, Ang II inhibits the actions of insulin in vascular and skeletal muscle, by interfering with phosphatidylinositol 3-kinase (PI3K) and its downstream signaling pathways through protein kinase B (PkB, also known as Akt). This inhibitory action is mediated, in part, by oxidative stress [12,13].

The RAS system is a pathway that is importantly regulated by peroxisome proliferator-activated receptors (PPARs). PPARs belong to the nuclear family of ligand activated transcription factors and comprise three different isoforms, PPAR-α, PPAR-β/δ, and PPAR-γ [7]., Our group had previously demonstrated the cardioprotective effects of PPAR-α stimulation (by fibrates) by reducing oxidative stress during myocardial ischemia [7,11,14,15]. Moreover, fenofibrate, a PPAR-α agonist, is beneficial in MetS, type 2 diabetes and cardiovascular diseases [11].

Therefore, our aim was to evaluate the effect of fenofibrate treatment on the myocardic Ang II/AT1 signaling pathway and its relationship with oxidative stress and myocardial insulin resistance in MetS rats under ischemic conditions.

## 2. Results

Table 1 shows the fasting basal characteristics of the experimental animals. Sucrose fed animals developed MetS characterized by hypertension, central adiposity, hyperinsulinemia and insulin resistance (IR). Levels of tryglicerides and non-high density lipoprotein cholesterol (non-HDL-C) were significantly higher in the MetS group than in the control (CT) group; furthermore, MetS rats had low levels of HDL-C.

None of the groups showed a significant difference in the glucose, or total cholesterol levels and neither body weight. In the CT group, fenofibrate treatment significantly reduced the concentration of non-HDL-C; however, in MetS animals fenofibrate therapy resulted in a reduction of triglycerides and non-HDL-C levels. No changes were observed in body weight, intra-abdominal fat, blood pressure, glucose, total cholesterol and HDL-C content between the CT and MetS fenofibrate-treated groups. Fenofibrate therapy significantly reduced insulin concentration in MetS rats and restored insulin resistance index (HOMA-IR). In the CT group, fenofibrate-administration did not alter significantly any parameters.

**Figure 1:**
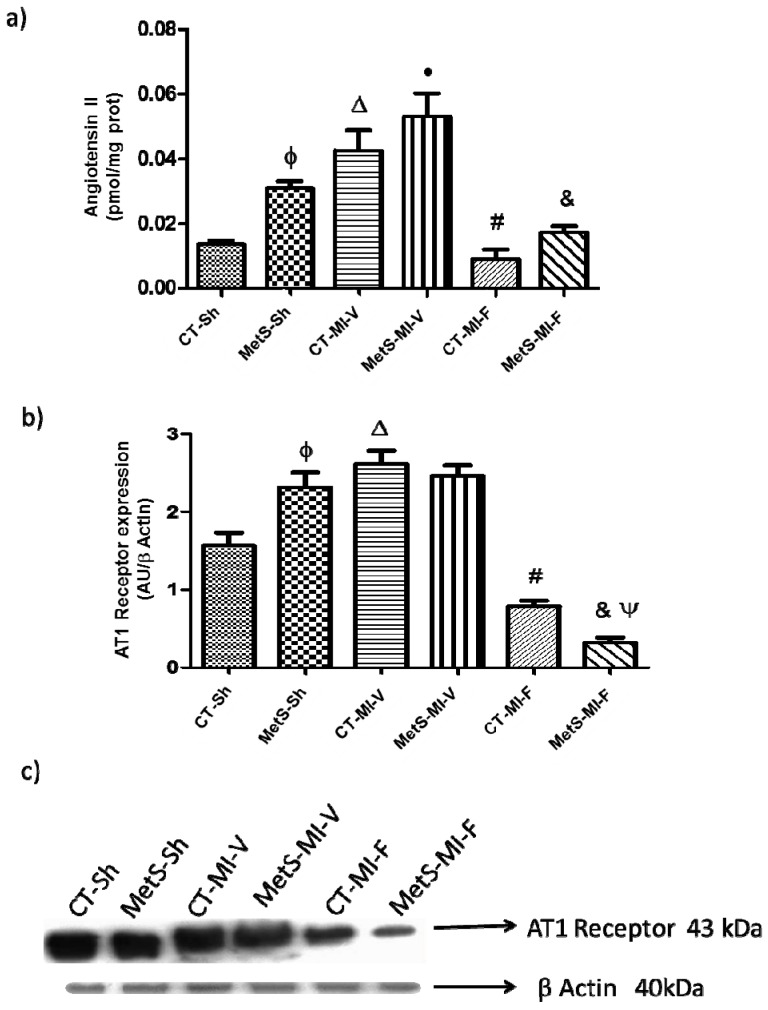
Effect of fenofibrate on angiotensin II (Ang II) concentration and the expression of AT1 receptor. (**a**) Ang II concentration was evaluated in the left ventricles from control (CT) and metabolic syndrome (MetS) rats subjected to sham- (Sh-) or myocardial infarction (MI) and treated two weeks with either vehicle (V) or fenofibrate (F); (**b**) AT1 protein expression, Arbitrary Units (AU); (**c**) Representative western blot analysis. Data represent mean ± SEM (*n* = 5 per group). φ *p* < 0.05 vs. CT-Sh; Δ *p* < 0.05 vs. CT-Sh; • *p* < 0.05 vs. MetS-Sh; # *p* < 0.05 vs. CT-MI-V; & *p* < 0.05 vs. MetS-MI-V; Ψ *p* < 0.05 vs. CT-MI-F. Analysis of variance-Tukey.

Figure 1a,b show the concentrations of Ang II and AT1 protein expression, respectively, in homogenate of the left ventricles from the different experimental groups. As was expected, Ang II levels were higher in MetS rats compared to CT rats. When hearts were under ischemic conditions, Ang II levels increased in CT and there was a further increase in MetS rats; however, fenofibrate treatment significantly diminished Ang II concentrations in both groups. AT1 expression was higher in MetS rats compared to CT rats (Figure 1b,c). Ischemia promoted an increase in AT1 expression in CT group, while is expression remained unchanged in hearts from MetS. Fenofibrate treatment significantly diminished AT1 expression in both groups although this effect was more evident in MetS rats (Figure 1b,c).

**Figure 2:**
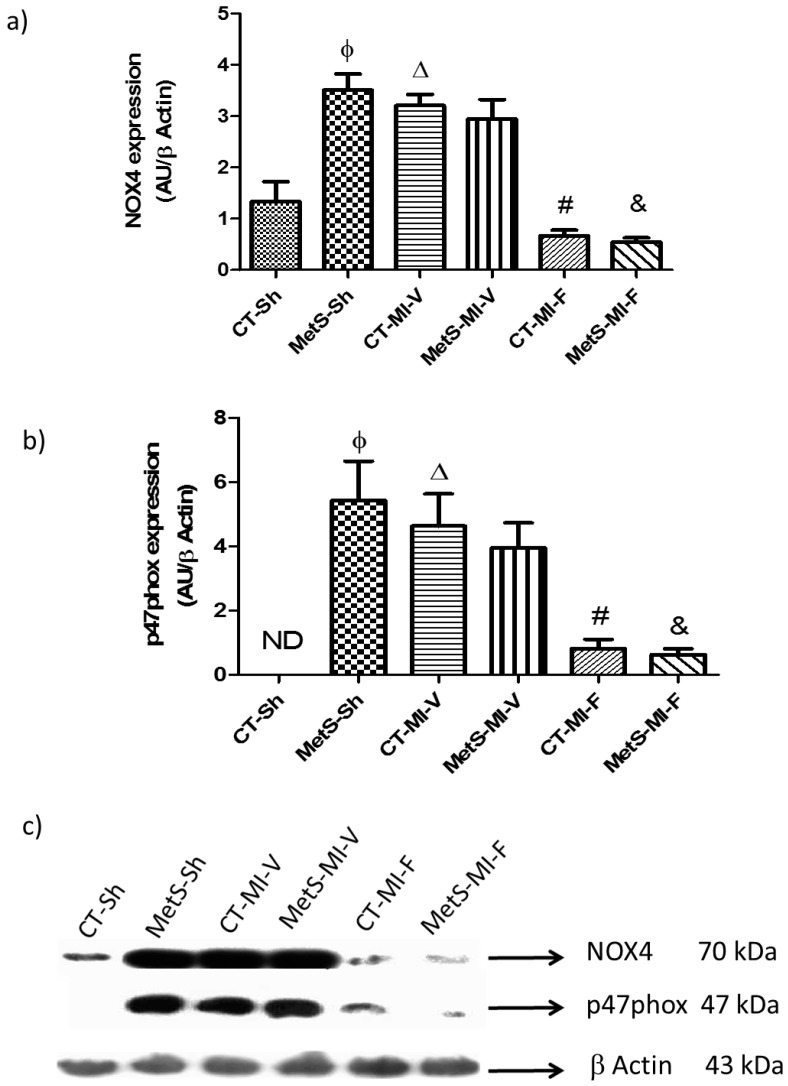
Effect of fenofibrate administration on myocardial nicotinamide adenine dinucleotide phosphate form (NADPH) oxidase (NOX4) (**a**) and p47phox (**b**) protein expression, in control and MetS rats under ischemic conditions; (**c**) Representative western blot analysis. ND: Not determined. Data represent mean ± SEM (*n* = 5 per group). φ *p* < 0.05 vs. CT-Sh; Δ *p* < 0.05 vs. CT-Sh; # *p* < 0.05 vs. CT-MI-V; & *p* < 0.05 vs. MetS-MI-V. Analysis of variance-Tukey.

We also evaluated the effect of fenofibrate administration on myocardial NOX4 expression and its main regulator (p47phox). These proteins are targets of the Ang II/AT1 signaling pathway. The results showed a statistically significant increase in the expression of NOX4 and p47phox in the MetS vehicle-treated group when compared to the corresponding CT group (Figure 2a–c). When the hearts were subjected to ischemia, NOX4 and p47phox were increased in the CT groups. Instead, their expression did not change in MetS rats. The administration of fenofibrate significantly decreased NOX4 and p47phox expression in the same proportion in both experimental groups (Figure 2).

**Figure 3:**
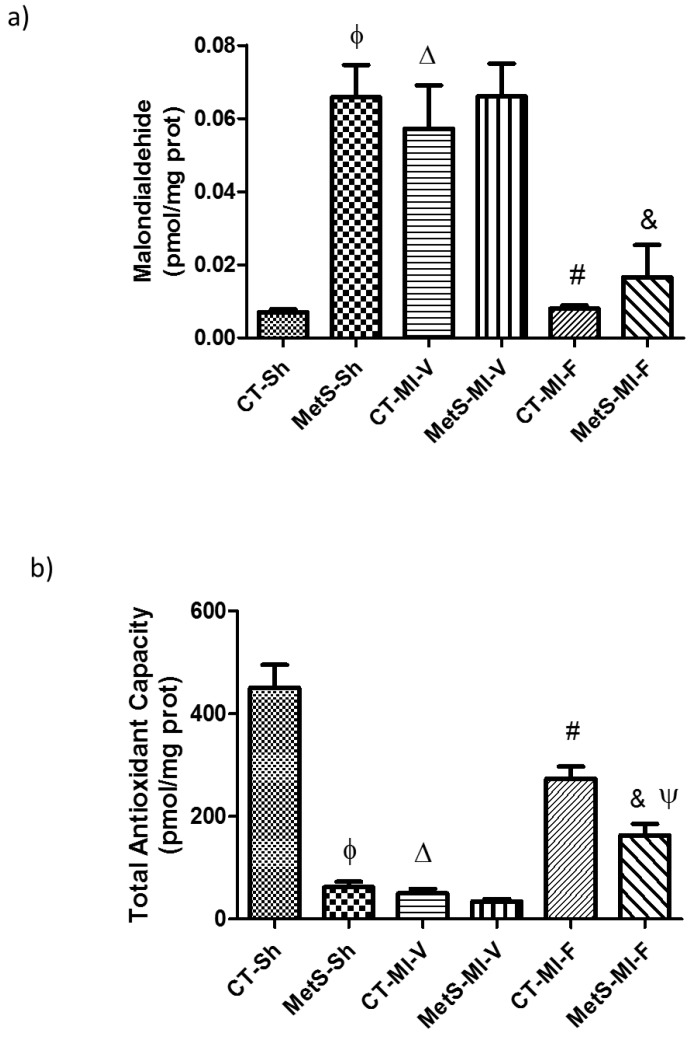
Effect of fenofibrate on malondialdehyde concentration (**a**) and total antioxidant capacity (**b**). Data represent mean ± SEM (*n* = 5 per group). φ *p* < 0.05 vs. CT-Sh; Δ *p* < 0.05 vs. CT-Sh; # *p* < 0.05 vs. CT-MI-V; & *p* < 0.05 vs. MetS-MI-V; Ψ *p* < 0.05 vs. CT-MI-F. Analysis of variance-Tukey.

To assess myocardial oxidative stress in experimental groups, we determined the total antioxidant capacity (TAC) and malondialdehide (MDA) levels and the expression of some antioxidant enzymes. We observed that in nonischemic hearts from the MetS group there was a higher MDA level compared to CT group (Figure 3a). There was also a lower antioxidant capacity compared to CT group (Figure 3b). Ischemia significantly increased MDA concentration and decreased TAC in C-vehicle-treated animals, while in MetS groups these variables remained constant. Fenofibrate treatment improved TAC and significantly decreased the MDA levels in both experimental groups (Figure 3a,b).

**Figure 4:**
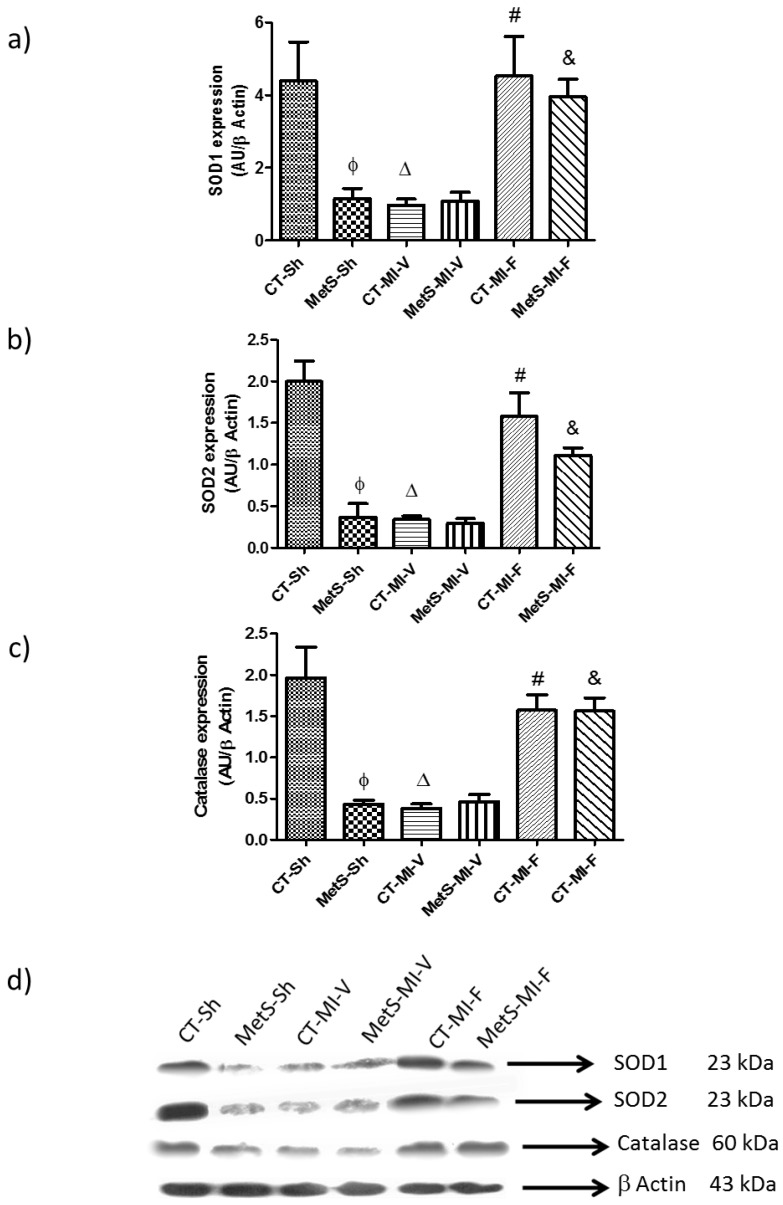
Expression of cardiac superoxide dismutase (SOD)1, SOD2 and catalase. Antioxidant enzymes were evaluated in the left ventricles from control and metabolic syndrome rats subjected to sham- or myocardial infarction and treated two weeks with either vehicle or fenofibrate. (**a**) SOD1 protein expression; (**b**) SOD2 protein expression; (**c**) catalase protein expression; (**d**) Representative immunoblot. Data represent mean ± SEM (*n* = 5 per group). φ *p* < 0.05 vs. CT-Sh; Δ *p* < 0.05 vs. CT-Sh; # *p* < 0.05 vs. CT-MI-V; & *p* < 0.05 vs. MetS-MI-V. Analysis of variance-Tukey.

The data in Figure 4a–c show the expression of superoxide dismutase (SOD)1, SOD2 and catalase in MetS rats. The expression of these enzymes was reduced when compared to CT rats. Under ischemic conditions, the expression of all of the antioxidant enzymes was significantly reduced in CT-vehicle treated compared to the MetS rats. Fenofibrate was able to prevent the decline in the expression of SOD1, SOD2 and catalase in both, CT and MetS animals.

**Figure 5:**
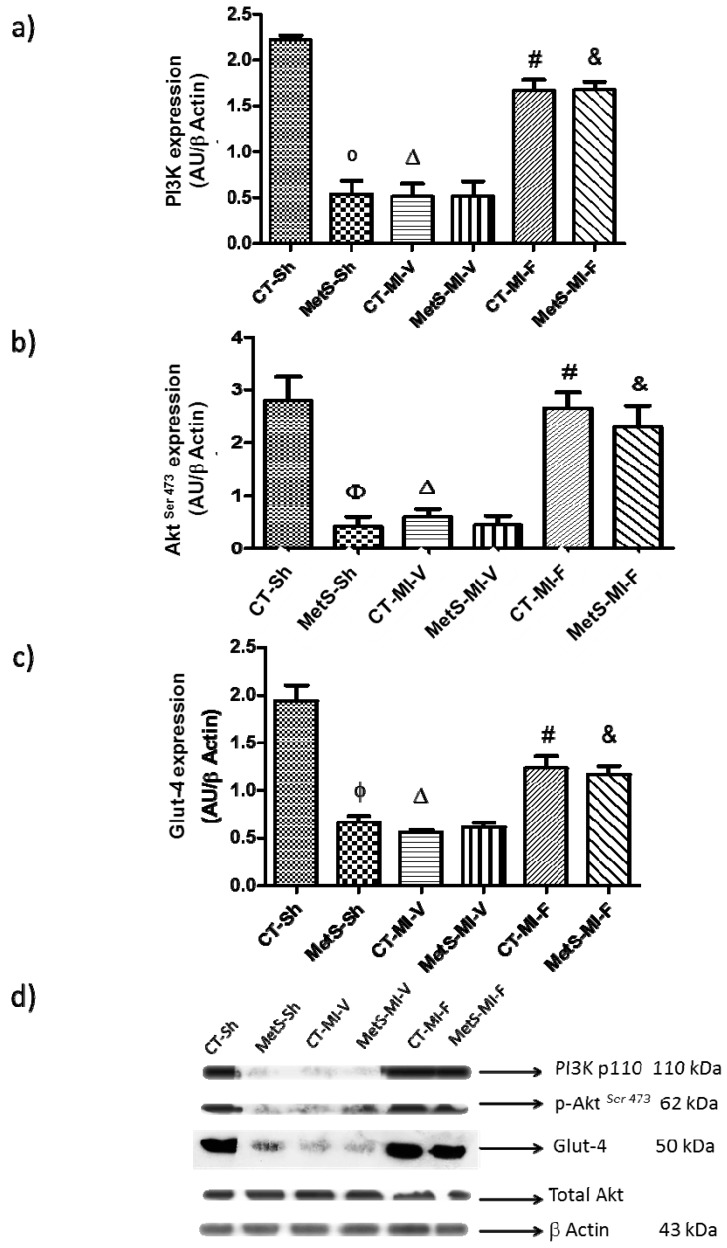
Fenofibrate treatment improves myocardial insulin resistance in MetS rats. Expression was evaluated by western blot in the myocardial ischemic area from sham, MI-V and MI-F groups. (**a**) PI3K p110α subunit; (**b**) p-Akt^Ser473^; (**c**) Glut-4 protein expression; (**d**) representative immunoblot. Data represent mean ± SEM (*n* = 5 per group). φ *p* < 0.05 vs. CT-Sh; Δ *p* < 0.05 vs. CT-Sh; # *p* < 0.05 vs. CT-MI-V; & *p* < 0.05 vs. MetS-MI-V. Analysis of variance-Tukey.

Next, we investigated whether fenofibrate-induced variations in Ang II and AT1 expression could be associated with the presence of myocardial insulin resistance. As expected, under basal conditions MetS hearts had insulin resistance evidenced by a lower expression of the PI3K p110α subunit, p-Akt^Ser473^ and Glut-4 compared to CT hearts (Figure 5a–c, respectively). Ischemia inhibited insulin signaling in hearts from CT-vehicle-treated by decreasing the expression of PI3K p110α subunit, p-Akt^Ser473^ and Glut-4. However, the expression of these components of the insulin pathway remained unchanged in hearts from MetS. Fenofibrate treatment restored the insulin sensitivity in CT and MetS rats.

**Figure 6:**
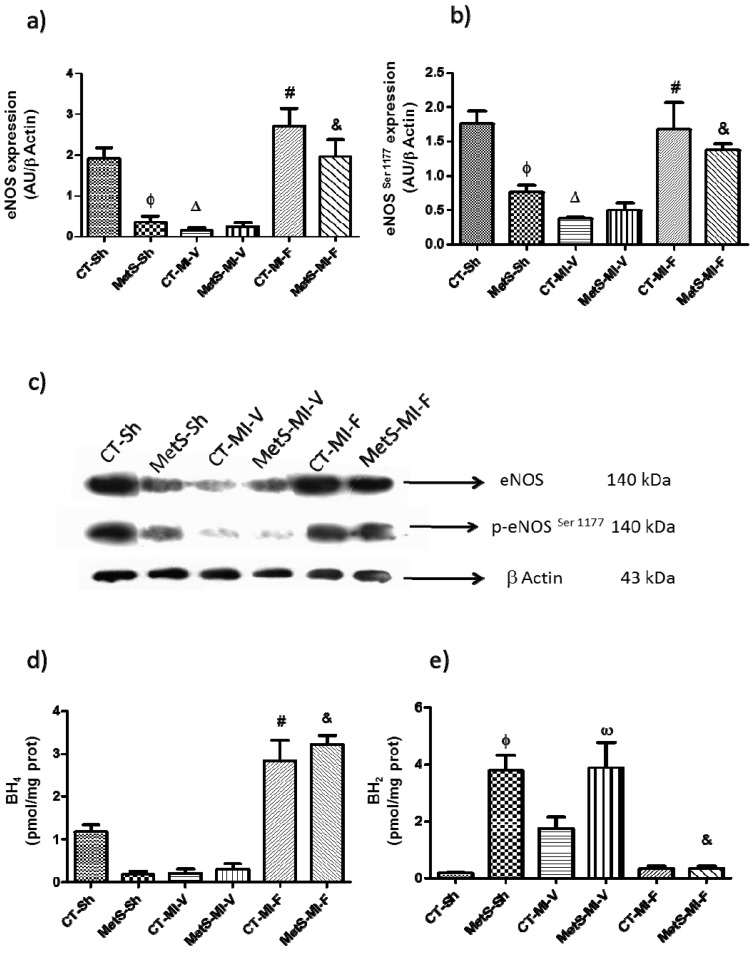
Effect of fenofibrate on myocardial expression of eNOS and biopterin concentrations. (**a**) Expression of eNOS; (**b**) Expression of p-eNOS^Ser1177^; (**c**) Representative western blot; (**d**) Biopterin levels; (**e**) Oxidized form of biopterin. Protein expression and biopterin concentrations were evaluated in the left ventricles from control (CT) and metabolic syndrome (MetS) rats subjected to sham- (Sh-) or myocardial infarction and treated two weeks with either vehicle (V) or fenofibrate (F). Data represent mean ± SEM (*n* = 5 per group). φ *p* < 0.05 vs. CT-Sh; Δ *p* < 0.05 vs. CT-Sh; # *p* < 0.05 vs. CT-MI-V; & *p* < 0.05 vs. MetS-MI-V; ω *p* < 0.05 vs. CT-MI-V. Analysis of variance-Tukey.

We also evaluated the expression and activity of eNOS in all of the experimental groups because it is critically regulated by insulin and it plays a major role in heart function. Our data show that eNOS and p-eNOS^Ser1177^ expression were significantly lower in MetS rats compared to the corresponding CT group (Figure 6a,b). eNOS and p-eNOS^Ser1177^ expression diminished in the left ventricular ischemic zone of CT-vehicle treated rats; however, the levels of these two isoforms were not modified in the hearts from MetS rats. The administration of fenofibrate significantly increased the expression of eNOS and p-eNOS^Ser1177^ levels in both groups (Figure 6a–c).

Due to the vascular relevance of BH_4_, we evaluated if concentrations of BH_4_ were modified by the oxidative stress present in MetS rats and under ischemic conditions. Figure 6d shows that under basal conditions, CT hearts had higher BH_4_ concentrations compared to MetS hearts. In CT vehicle-treated animals, BH_4_ levels decreased under ischemic conditions; nevertheless, in the MetS vehicle-treated group, BH_4_ levels were not modified. The concentrations of oxidized form of biopterin (BH_2_) showed the opposite effect (Figure 6e). The pharmacological treatment with fenofibrate was associated with a significant increase of BH_4_ and the consequent decrease of BH_2_ concentrations in both groups.

**Figure 7:**
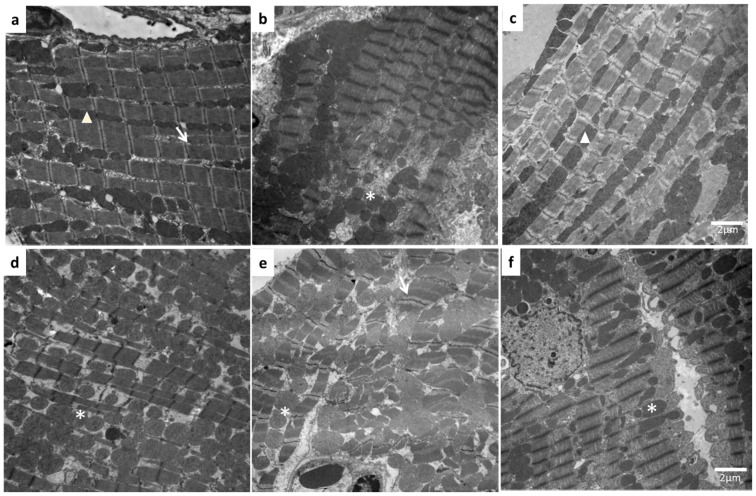
Fenofibrate attenuated myocardial ultrastructural damage in MetS and myocardial infarcted rats. CT-Sh (**a**); CT-MI-V (**b**); CT-MI-F (**c**); MetS-Sh (**d**); MetS-MI-V (**e**); MetS-MI-F (**f**). Details by electron microscopy in sarcomere. Mitochondria arranged in rows (Δ); I band (→); swollen mitochondria (*). Magnification 8000×, scale bar represents 2 μm. Images are representative of six different experiments.

Finally, to determine whether fenofibrate exerts any effect on myocardial histology, we evaluated the ultrastructure of the ischemic myocardial area. We observed that although CT-Sh rats maintained the typical structure of a myocardial fiber (sarcomere and band I) and an even distribution of mitochondria in rows; ischemic tissue from CT-MI-V showed a loss of tissue organization: there was separation of myocardial fibers and swelling of mitochondria (Figure 7a,b). MetS alone induces structural changes such as a loss of the striated pattern (an increase in the I band), swelling of mitochondria and a decrease in the density of the mitochondrial matrix. These structural changes are further marked in MetS-MI-V animals (Figure 7e). Treatment with fenofibrate attenuated ischemia-induced tissue injury in MetS and CT rats, but the improvement was more evident in the CT group (Figure 7f,c).

## 3. Discussion

MetS is a cluster of risk factors that leads to cardiovascular diseases. MetS doubles the incidence of coronary artery disease; it increases the progression of the atheromatous plaque and is associated to heart failure [16]. RAS dysregulation mediates many of the changes in MetS and myocardial infarction (MI) and there is a link between the Ang II/AT1 signaling pathway and alterations in cardiac energy metabolism, cardiac insulin resistance, and the development of heart failure.

Treatment with the dyslipidemia corrector, anti-inflammatory, endothelial protective, antioxidant, and antithrombotic agent fenofibrate significantly reduces nonfatal myocardial infarction and coronary revascularization in subjects with diabetes and MetS [17]. Beneficial effects of fenofibrate might be explained by its triglyceride-lowering effect and by PPAR-α activation [11,18]. PPAR-α stimulation protects the heart during ischemia by regulating myocardial metabolism, increasing antioxidant defenses, decreasing ROS and modifying RAS participation [14,15,16,17,18,19,20].

However, there are very few studies showing the relationship between RAS activation, local oxidative stress and myocardial insulin resistance. Thus, here we evaluated whether sub-chronic treatment with fenofibrate inhibits the activation of Ang II/AT1 pathway after MI. MI is associated with myocardial oxidative stress which inhibits the insulin signaling cascade in hearts of MetS rats.

MetS rats had hypertension and displayed lipid profile disturbances. There was not a statistically significant difference in weight between the CT and MetS rats even though the diet of the sucrose-fed rats was hypercaloric; however, the MetS animals showed increased central adiposity, which is one of the characteristics of MetS model (Table 1). The increase in abdominal fat was most likely accompanied by a decrease in muscle mass because body weight did not significantly increase. In our model, we have not determined a difference in muscle mass between the CT and MetS rats, but sucrose fed animals have been shown to consume less solid food, which means less protein and mineral intake.

Administration of fenofibrate significantly reduced triglycerides and non-HDL-C levels in MetS animals. These results agree with previous studies that demonstrated the anti-dyslipidemic effect of fenofibrate by changing low density lipoprotein (LDL) particle morphology and reducing triglycerides in humans and animal models [21,22,23].

Insulin was increased in MetS rats and there was insulin resistance indicated by HOMA-IR. Insulin resistance and the consequent hyperinsulinemia are signs of MetS involved in the development of cardiovascular diseases. Our results show that fenofibrate therapy significantly reduced insulin concentration in MetS rats and restored HOMA-IR without changing glucose concentration. In the CT group, fenofibrate-administration did not alter any of these parameters. There was a statistically significant decrease in fasting plasma insulin levels when triglycerides were reduced. This result is consistent with studies that have shown that marked hypertriglyceridemia is associated with insulin resistance and that fibrates improve glucose tolerance or increase insulin sensitivity in humans and rodents.

The beneficial effect on insulin sensitivity may be due to a decrease in body weight, adiposity and free fatty acids (FFA). It might also be caused by facilitated fatty acid transport to mitochondria and stimulation of their oxidative degradation in muscle [24]. Additionally, when FFA are decreased by fenofibrate treatment, inflammation due to MetS which is mediated by the activation of Toll-like receptors by FFA might decrease. An inflammatory state might contribute to the presence of insulin resistance and oxidative stress [25].

Despite recent reports on the favorable effect of fenofibrate on body weight, HDL-C concentrations and systolic blood pressure in various models of obesity and hypertension [18,26,27], we did not find a significant change in these parameters in our MetS-F group.

Ang II might be the link between insulin resistance, oxidative stress and cardiac metabolism; moreover, several studies have demonstrated increases in systemic and tissue specific Ang II levels in humans and in animal models of hypertension, obesity and MetS [7,16]. As expected, Ang II levels and AT1 protein expression were increased in the left ventricles from MetS rats (Figure 1a,b). After the ischemic insult, Ang II concentrations were significantly increased in CT and MetS hearts. We found an increase in AT1 expression in the CT group while it remained constant in MetS rats (Figure 1b,c). The fenofibrate-treatment significantly diminished Ang II concentrations and AT1 expression in both groups. This result had been previously reported in different models by other authors [28,29,30]. In another hand, Ang II is also an inflammatory mediator that activates NF-κB [25]. Fenofibrate therapy diminishes the amount of myocardial Ang II and therefore, we cannot exclude an inflammatory effect similar to the one previously reported by our group [31].

The interaction of Ang II/AT1 stimulates the enzymatic activity of NOX in endothelial and smooth muscle cells. After assembly of its subunits, NOX catalyzes the reduction of molecular oxygen to superoxide which is converted to H_2_O_2_ by SOD. H_2_O_2_ is, in turn, converted to water by catalase or glutathione peroxidase [32,33]. Therefore, in this study, the expression of the subunits of membrane NOX-4 and of the cytosolic p47phox NOX were determined. Our results show that there is an overexpression of the NOX subunits under basal conditions in hearts from the MetS rats (Figure 2). Other authors had already reported that the expression and activity of NOX is enhanced in the hearts of obese Zucker rats, leptin-deficient *ob/ob* mice and high fat-fed rats [16]. The expression of both subunits was significantly diminished with fenofibrate. A similar effect was observed by Calkin et al. [34] in diabetic mice treated with gemfibrozil. Furthermore, previous experiments also showed a reduction of NOX activity when using an agonist for PPAR-α activity [35,36].

We also investigated the association between NOX expression and oxidative stress. Our results show that the presence of MetS increases MDA and diminishes TAC levels (Figure 3a,b). Under ischemic conditions TAC diminished and MDA increased in the CT group while in the left ventricles from MetS animals these variables remained constant. The fenofibrate treatment significantly improved TAC and diminished MDA production in both groups. These results are similar to the ones previously obtained in our laboratory where we demonstrated that PPAR-α stimulation by clofibrate diminished the production of ROS and lipid peroxidation. TAC was significantly restored in the clofibrate treated group [14].

SOD1, SOD2 and catalase are important components of the antioxidant defense system protecting the heart against ischemic damage. Therefore, we evaluated their expression in our experimental animal model in which cardiac antioxidant enzymes are reduced [6]. Nevertheless, we found that in MetS, MI, and their combination, the antioxidant reserves are diminished. The exposure to oxidative stress induced by the activation of NOX might exhaust the antioxidant capacity of the heart. Fenofibrate treatment prevents the diminution in the expression of SOD1, SOD2 and catalase in MetS animals and in the CT hearts exposed to ischemic conditions (Figure 4a–c). Our results support that the cardioprotective effect is due to a decrease in the activation of Ang II/AT1/NOX pathway [37,38]. Other authors have described the importance of PPAR-α on the expression of these enzymes [39,40]. Our results agree with those of Inoue et al. [36] who found that activation of PPAR-α by bezafibrate increases the genetic expression and the protein levels of SOD2 in endothelial cells. The use of natural and/or artificial antioxidants is a strategy for the treatment of abnormalities associated to MetS; antioxidants improve insulin sensitivity and protect against ischemia-induced damage [41,42,43].

RAS and insulin signaling in the heart constitute a complex network including multiple feedback loops and crosstalk. Ang II may function as a negative modulator of Glut-4 gene expression and may induce local insulin resistance accompanied by the development of a cardiac failure [44]. However, it might also decrease myocardial insulin signaling, representing a potential mechanism for the beneficial effect of high-fat diets that decrease heart failure in rodent models of pressure overload or post-myocardial infarction [1].

As expected, MetS hearts under basal conditions had insulin resistance evidenced as a lower expression of the PI3K p110α subunit, p-Akt^Ser473^ and Glut-4 compared to CT hearts. Ischemia inhibited insulin signaling in hearts from CT-vehicle-treated rats by decreasing the expression of PI3K p110α subunit, p-Akt^Ser473^ and Glut-4. However, the expression of these components of the insulin pathway remained unchanged in hearts from MetS (Figure 5a–c).

Our results show that pharmacological treatment was associated with activation of p-Akt^Ser473^ by PI3K leading to increases in the myocardial protein expression of Glut-4. They agree with those of Ide et al. [45], Yang et al. [46] also demonstrated that fenofibrate treatment has a protective effect activating the PI3K/Akt pathway in mice subjected to renal ischemia-reperfusion. Taken together, our results provided strong evidence that oxidative stress impairs myocardial insulin action. The beneficial effect of fenofibrate by restoring the myocardial insulin signaling may increase glucose utilization.

Finally, since eNOS expression and activity is regulated by the insulin signaling pathway (PI3K/p-Akt^Ser473^) and is impaired in myocardial ischemia, we investigated whether the restoration of insulin sensitivity by fenofibrate had an effect on the expression of eNOS and its phosphorylated isoform (p-eNOS^Ser1177^). We observed that eNOS and p-eNOS^Ser1177^ expression were significantly lower in MetS hearts. The ischemic insult induced the down-regulation of the eNOS isoforms in CT-V group while in hearts from MetS rats they remained unchanged (Figure 6a–c). The pre-treatment with fenofibrate induced eNOS and p-eNOS^Ser117^ protein expression in both, CT and MetS rats. Our results suggest that the effect of fenofibrate on eNOS in rats with MetS under MI conditions is mediated by genomic actions through PPAR-α activation (eNOS expression) and by non-genomic actions. Non-genomic effects involve activation of the PI3K/ p-Akt^Ser473^ signaling pathway (phosphorylation of eNOS). These results are in agreement with previous studies [47].

BH_4_ is a cofactor necessary for eNOS coupling favoring the formation of NO and reducing the production of superoxide radicals [11,48]. The oxidant environment present in MetS and the MI condition was accompanied by a reduction in the BH_4_ concentration and the consequent increase in the concentration of BH_2_. Fenofibrate treatment favored the production of BH_4_ and reduced BH_2_ in the left ventricle from both groups of rats (Figure 6d,e). This result is similar to that obtained by Dumitrescu et al. [49]. Thus, the resultant increase in the production and bioavailability of NO mediates coronary vasodilation, myocardial substrate flexibility and energy homeostasis.

On the other hand, increased oxidative stress (present in MetS and in MI) may also affect tissue structure. Ultra-structural alterations were demonstrated by electron microscopy in the left ventricles even 60 min after ligation of the coronary artery (Figure 7). The changes described for CT-MI-V group were further increased in the tissues from MetS-MI-V animals: separation of myofibrils was increased and their borders irregular and indistinct and mitochondrial swelling (Figure 7b,e). Pharmacological treatment with fenofibrate was able to attenuate MI-induced cardiac structure damage (Figure 7c,). We think that this is the result of actions promoted by fenofibrate treatment, such as favors an antioxidant environment as a consequence of a reduction of the Ang II/AT1/NOX4 signaling pathway, reestablishing the cardiac insulin signaling pathway. This mechanism also improves NO production and bioavailability. The protective effect shown, might probably be translated in a better cardiac function. Further studies would be needed to prove this point.

One of our aims in this study was to evaluate if the myocardial infarction could enhance the damage in the MetS associated variables analyzed in a similar way as in the diabetes model [11]. However, our results show that the presence of MetS per se causes damage to the heart and most of our results indicate that the induction of ischemia in MetS animals, does not exert a synergistic effect on some variables associated to the pathology. Thus, heart protective mechanisms by fenofibrate might be established during the two weeks of the treatment, and be present before the hearts are put into ischemic conditions in MetS animals.

## 4. Materials and Methods

### 4.1. Experimental Animals

The study was approved by the animal ethics committee of our Institution (No. 14-862). Thirty six Wistar male rats (raised in our Institution) weighing 50 ± 5 g, were divided into two groups: group 1, control rats , given tap water for drinking, and group 2, MetS rats, receiving 30% sugar in their drinking water during 24 weeks.

All animals were fed with standard chow diet (LabDiet 5001, PMI Nutrition International, LLC. Brentwood, MO, USA) ad libitum, which provides 14.63 kJ/g, with 23% protein, 4.5% fat, 65% carbohydrate, 0.39% sodium and 0.64% chloride. The animals were kept under controlled temperature and a 12:12-h light-dark cycle.

At the end of the 24 weeks, animals from each experimental group (CT and MetS) were divided to receive one of the sub-chronic (two weeks) oral treatments: (a) vehicle (NaCl 0.9%) or (b) fenofibrate (100 mg/kg/day). The rats were weighed and their systolic arterial blood pressure was measured by the tail cuff method [7]. At the end of the treatment, the animals were assigned either to sham-operation or myocardial infarction for 60 min.

### 4.2. Myocardial Infarction

Anesthesia was induced with ketamine hydrochloride (100 mg/kg, intramuscularly) and xylazine hydrochloride (20 mg/kg, intramuscularly), both drugs were provided by PiSA Farmaceutica^®^ (Guadalajara, Jal., Mexico). The rats were artificially ventilated (70 strokes per minute tidal volume: 8–10 mL/kg) as previously reported [8] and MI was achieved by occluding the left anterior descending coronary artery for 60 min. After acute MI, the heart was cut out and the ischemic area was separated and stored at −70 °C for later analysis. Additionally, the visceral white adipose tissue was removed and weighed.

### 4.3. Biochemical Serum Measurement

To determine basal characteristics of the experimental animals, after overnight fasting, the rats were decapitated and their blood was collected; serum was isolated by centrifugation and stored at –70 °C until needed. The baseline fasting measurements of glucose, total cholesterol, high-density lipoprotein cholesterol, non HDL-C and triglycerides were carried out with commercial enzymatic kits (RANDOX Laboratories Ltd., Crumlin, County Antrim, UK). Serum insulin levels were measured using a rat-specific insulin radioimmunoassay kit (Linco Research, Inc., Saint Charles, MO, USA). Insulin resistance was estimated from the homeostasis model (HOMA-IR) [41].

### 4.4. Measurement of Total Antioxidant Capacity

TAC was determined following the method as previously reported by Ibarra-Lara et al. [15] based on the reduction of Cu^2+^ to Cu^1+^.

### 4.5. Quantification of Ang II, Malondialdehyde, Tetrahydrobiopterin and Dihydrobiopterin

Myocardial Ang II, MDA, BH_2_ and BH_4_, production was evaluated by capillary zone electrophoresis (CZE), according to the methods described previously [15]. Data are expressed as pmol of metabolite per mg of protein.

### 4.6. Western Blotting

The frozen myocardial ischemic area was homogenized with a polytron (model PT-MR2100; Kinematica AG, Lucerne, Switzerland) (25% *w*/*v*) in a lysis buffer pH = 7.4 (250 mM Tris-HCl, 2.5 mM EDTA) and a mixture of protease inhibitors (10 μg/mL leupeptine and 20 μg/mL aprotinin) at −4 °C [8]. A total 100 μg protein were separated on a SDS-PAGE (12% bis-acrilamide-laemmli gel) and transferred to a polyvinylidene difluoride (PVDF) membrane. The blots were blocked for 3 h at room temperature with Tris buffer solution (TBS) containing 5% nonfat dry milk and 0.05% Tween 20. The membranes were incubated overnight at 4 °C with rabbit primary polyclonal antibodies from Santa Cruz Biotechnology (Santa Cruz, CA, USA). Primary antibodies used were: AT1 receptor, NOX4, p47phox, SOD-1, SOD-2, catalase, PI3K p110α subunit, Akt, phospho-Akt (Ser 473), Glut-4, eNOS and phospho-eNOS (Ser 1177). Then, the membranes were incubated for 2 h at room temperature with a horseradish peroxidase-labeled antibody from Bio-Rad (Bio-Rad Laboratories, Inc., Hercules, CA, USA). All blots were incubated with β-actin as control. After incubation, the blots were visualized using a chemiluminescence kit (Immobilon Western, Millipore, MA, USA). Images from each film were acquired by GS-800 densitometer (including Quantity One software from Bio-Rad Laboratories, Inc., Hercules, CA, USA). The values of each band density are expressed as arbitrary units (AU).

### 4.7. Structural Analysis by Electron Microscopy

Electron microscopy was done by the method previously reported by Ibarra-Lara et al. [20]. Ischemic myocardial areas from all experimental groups, were fixed with 2.5% glutaraldehyde for 1 h and then stored in 0.1 M cacodylate buffer. After postfixing in 1% osmium tetroxide in 0.1 M cacodylate buffer, the samples were dehydrated in a graded series of ethanol and embedded in EPON 812 (Electron Microscopy Sciences, Hatfield, PA, USA). Ultrathin sections (approximately 60 nm thick) were cut using an Ultracut microtome (Leica Biosystems, NuBloch, Germany) and mounted onto copper grids. Sections were contrasted with uranyl acetate and lead citrate before evaluation with a JEM-1011 (JEOL Ltd., Tokyo, Japan) at 60 kV. Random pictures of 2–3 myocardial ischemic areas were taken from three rats per group using 8000× magnification.

### 4.8. Statistical Analysis

Results are expressed as the mean ± standard error of the mean (SEM). For multiple comparisons, we applied one-way analysis of variance (ANOVA) followed by post hoc test (Tukey or Student *t* test) using Graph Pad Prism software version 5.03 (GraphPad Software, La Jolla, CA, USA). Differences were considered significant when the *p* value was <0.05.

## 5. Conclusions

Although many mechanisms might participate in the cardioprotective effect of fenofibrate such as its anti-inflammatory effect, our results demonstrate that activation of the Ang II/AT1 pathway leads to oxidative stress and the presence of myocardial insulin resistance in MetS rats. Therefore, the fenofibrate treatment in animals having MetS favors an antioxidant environment in the heart that is a consequence of a reduction of the Ang II/AT1/NOX signaling pathway. This, in turn, reestablishes the cardiac insulin signaling pathway (PI3K/Akt/Glut-4/eNOS) thus allowing an optimization of the cardiac metabolism, an improved vasodilator function and preserving cardiac structure during ischemia in the heart.

## Figures and Tables

**Table 1:** Effects of fenofibrate on body characteristics and baseline fasting biochemical parameters from control (CT) and metabolic syndrome (MetS) rats.

	CT-V	CT-F	MetS-V	MetS-F
Body weight (g)	522.0 ± 12.4	463.5 ± 28.9	524.9 ± 23.9	490.4 ± 17.1
Visceral fat (g)	6.2 ± 0.8	4.4 ± 0.9	12.0 ± 0.6 ^a^	12.1 ± 1.4
Blood pressure (mmHg)	97.3 ± 7.2	90.7 ± 2.6	142.1 ± 1.8 ^a^	142.7 ± 16.8 ^a^
Glucose (mg/dL)	105.2 ± 9.8	97.8 ± 6.1	115.2 ± 17.8	102.6 ± 6.3
Insulin (ng/mL)	0.08 ± 0.04	0.09 ± 0.03	0.29 ± 0.04 ^a^	0.10 ± 0.05 ^b^
HOMA-IR	0.81 ± 0.32	1.15 ± 0.38	3.52 ± 0.6 ^a^	1.2 ± 0.53 ^b^
Total cholesterol (mg/dL)	62.4 ± 7.5	48.8 ± 1.9	57.6 ± 1.7	40.6 ± 5.3
HDL-C (mg/dL)	36.9 ± 5.8	36.7 ± 1.6	23.7 ± 4.3 ^a^	25.6 ± 6.4
Non-HDL-C (mg/dL)	21.7 ± 3.2	12.1 ± 2.2 ^b^	30.4 ± 1.6^a^	11.2 ± 3.1 ^b^
Triglycerides (mg/dL)	51.20 ± 21.3	20.8 ± 2.3	140.6 ± 25.2 ^a^	41.30 ± 13.2 ^b^

Values are mean ± standard error of the mean (SEM). CT-V: control vehicle-treated; CT-F: control fenofibrate-treated; MetS-V: metabolic syndrome vehicle-treated, MetS-F: metabolic syndrome fenofibrate-treated; HOMA-IR: Homeostatic model assessment of insulin resistance; HDL-C: high density lipoprotein cholesterol; *n* = 8; ^a^ *p* < 0.01 MetS vs. CT same treatment; ^b^ *p* < 0.05 against vehicle corresponding group.
